# Determination of Anthracene Derivatives in Baltic Amber Using SERS

**DOI:** 10.3390/s23042161

**Published:** 2023-02-14

**Authors:** Anna Kundalevich, Andrey Zyubin, Karina Matveeva, Ilia Samusev, Ivan Lyatun

**Affiliations:** 1 Research and Education Center “Fundamental and Applied Photonics. Nanophotonics”, Immanuel Kant Baltic Federal University, A. Nevskogo 14, 236016 Kaliningrad, Russia; 2 The International Research Center “X-ray Coherent Optics”, Immanuel Kant Baltic Federal University, A. Nevskogo 14, 236016 Kaliningrad, Russia

**Keywords:** Baltic amber, silver, nanoparticles, Raman, SERS

## Abstract

The article describes the results of Raman spectroscopy and SERS for the study of fluorescent components of Baltic amber via the extraction method. Using SERS, it was possible to confirm the presence of anthracene derivatives in amber: tetracene and benzanthracene. It has been shown that SERS methods are effective for the detection of aromatic compounds; they increase the registered Raman signal and make it possible to identify peaks characteristic of the compounds under study. By combining experimental methods with DFT simulations, anthracene derivatives were modeled and confirmed to be present in the structure of Baltic amber. A combination of the proposed methods can be used to distinguish between different types of amber and isolate the necessary amber components. The obtained results are promising for compiling spectral maps of ambers for their possible classification by their place of origin.

## 1. Introduction

Over the last decades, a large number of studies have been carried out on fossil resins, determining their age, analyzing inclusions in natural resin [1,2,3,4,5], determining the geographical and geological deposits [6,7], distinguishing real natural resins from fakes [8], and distinguishing different varieties [9,10,11,12,13,14]. To date, more than a hundred different fossil resins have been described [15]. Many resins contain terpenoid compounds that readily polymerize when exposed to light or air, resulting in the formation of hardened resinous masses that can also be observed on modern trees [16]. Cured resins are often highly resistant to many of the typical degradation mechanisms and usually persist in deposits. The fossilized form of plant resin is known as amber.

It is known that amber contains several chemical elements: carbon, hydrogen, and oxygen. However, in different types of amber, the percentage varies [17]. Amber is classified based on the chemical nature of the polymerized terpenoids that make up the macromolecular structure. For example, class I amber is divided into subgroups [16]. Class Ia includes polylabdanoid ambers containing succinic acid (Baltic amber). Class Ib also includes polylabdanoid ambers; however, specimens of this group lack succinic acid. Class Ic amber also does not contain succinic acid but is based on enantio-series labdanoid polymers.

In the study of amber, special attention is paid to its spectral properties. At present, nondestructive optical methods such as fluorescence and vibrational spectroscopy are used to study the chemical composition of amber and amber-like resins [18]. Since the intensity of fluorescence is proportional to the concentration of the fluorophore, it is possible to assess the degree of heterogeneity of a given substance in a sample. Depending on the type of amber particles, luminescence from bluish to green can be observed [19].

To study the optical and chemical properties of amber, vibrational spectroscopy methods are used to make it possible to conduct a qualitative assessment of the sample and compare the degree of destruction of amber after thermal aging, distinguish real amber from false, and determine the geographical origin of samples [20]. However, a detailed description of the chemical structure of amber is hampered by its non-crystallinity and poor solubility, so methods such as X-ray crystallography and nuclear magnetic resonance (NMR) spectroscopy in the solution phase are not suitable for analyzing the structure [21].

Vibrational spectroscopy methods (infrared and Raman spectroscopy) are the most suitable for the analysis of the chemical structure of amber and amber-like resins due to the fact that they can be used to obtain an accurate spectrum and correlate it with the chemical structure of amber. Infrared (IR) spectroscopy is by far the most used method for performing spectral analyses of amber and obtaining information about its structure and its changes [21,22,23,24], although relatively few works have been identified on both types of spectroscopies. Based on the methods of IR Fourier spectroscopy, the problems of differentiating amber types, identifying spectral features in the mid-frequency and high-frequency ranges for differentiating different types of amber, and comparing them with each other are solved [23,24,25,26,27]. The Raman spectroscopy technique is effective for the analysis of natural resins, allowing the accurate identification of their chemical structures and their changes [28,29,30]. Some of the latest papers are aimed at modeling the spectral components of amber [31], the study of the chemical composition of amber of various geographical origins [32], the study of the problems of amber oxidation [33], and changes in the structure of amber during thermal action applications [34]. However, this type of spectroscopy in the study of amber is less common in contrast to IR spectroscopy. In general, vibrational spectroscopy (FTIR and Raman) is generally used to determine the complex aromatic components [35] as well as the molecular vibrations that make up the molecular framework of natural resin [30].

The applied methods are based on the difference in the chemical structure of amber and amber-like resins [36,37,38,39,40]. Recently, the emphasis in research has also been on a combination of research techniques supplemented by mathematical modeling methods to identify differences between various ambers and amber-like resins [41,42,43,44]. Mathematical modeling can be used to establish the spectral assignment of communic acids, which are important isomers in the backbone of class I fossil resins, in order to distinguish amber from less mature resins [31].

However, there are two major drawbacks to the application of Raman spectroscopy, including inherently weak signals and autofluorescence interference, which make it not sensitive enough to detect low-concentration samples. To overcome the problem of the low efficiency of Raman scattering, surface-enhanced Raman spectroscopy (SERS) was developed, which is based on the enhanced scattering of an analyte of interest near a rough metal surface [45], a colloidal solution [46], or a rough electrode [47]. Another method for detecting small particles is the use of silver nanoparticles (AgNPs). The detection method based on SERS using a colloidal solution of silver makes it possible to identify micro- and nanoplastics in liquids [48]. Silver nanoparticles can be deposited on quartz glass and followed by SERS [49]. Silver nanoparticles also help to perform SERS mapping on silicon wafers [50]. To detect nanoplastics, a method was proposed using a bifunctional Ag nanowire membrane [51].

This paper proposes a technique that allows for the spectral identification of Baltic amber as well as its constituents. With the usage of silver nanoparticles, SERS techniques for the study of amber were successfully implemented. Amber has a complex structure, which, in its composition, has a large number of aromatic compounds. It has been suggested that extracting amber will help isolate some of the fluorescent groups that can be detected using SERS. It was also shown that, for the study of extracts of aromatic compounds, SERS methods are effective—increasing the detected signal by at least 30 times—and the only ones possible to use. Theoretical spectra of putative compounds in the structure of natural resin (naphthacene and benzo [a]anthracene) were calculated through DFT mathematical modeling. It was shown that these substances can be found in the composition of Baltic amber due to its extraction method. Based on the results of the work carried out, the prospects for the analysis of amber using the Raman spectroscopy method are shown.

## 2. Materials and Methods

**Figure 1:**
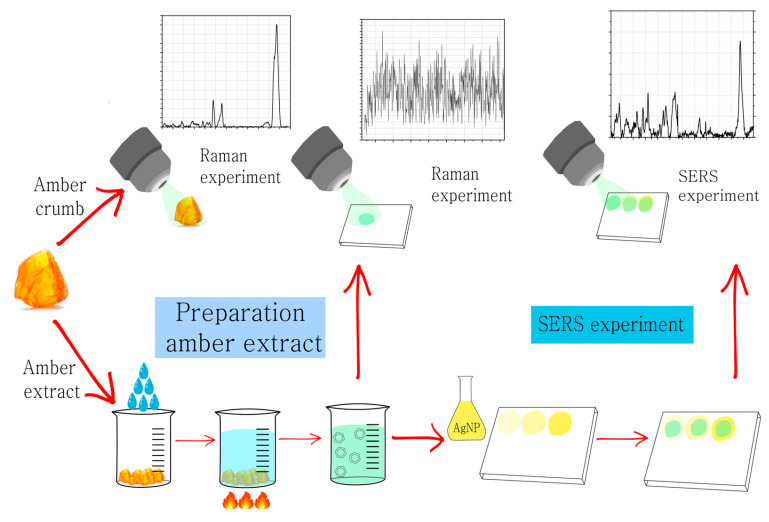
Conducting an experiment: Raman spectroscopy study of amber particles and amber extract, SERS study of amber extract.

A schematic illustration of the Raman and SERS experiment is shown in Figure 1.

### 2.1. Spectrophotometry Experiment

The amber absorption study was carried out using a Shimadzu-2600 spectrophotometer (Shimadzu, Kyoto, Japan) designed to obtain absorption, transmission, reflection, and energy spectra. The excitation was carried out in the wavelength range of 200–800 nm. Polished Baltic amber was used to study the absorption of amber. A total of 1081 g of amber was weighed on an ALC-210d4 (Aculab, Norwood, MA, USA) laboratory analytical scale, which was then placed in a 10 × 10 mm cuvette for spectrophotometry with transparent walls, and then, together with the sample, the cuvette was placed in the sample holder of the instrument. The sample spectrum was taken using the UVProbe program in “Spectrum” mode. After that, the obtained absorption spectrum of the sample was recorded in the txt format, which was further processed in the Origin program.

### 2.2. Raman Experiment

The Raman scattering spectra of amber and amber extract were studied using the research complex Centaur U HR (NanoscanTechnology, Moscow, Russia). The amber extract was prepared as follows: a certain amount of the amber fraction was weighed on electronic scales, after which the amber was placed in a tall glass with a volume of 50 mL. Using an Eppendorf Research+ automatic pipette, 40 mL of distilled water was measured and added to the amber. Next, the glasses were placed on an induction stove. Water with an amber fraction was brought to a boil. To minimize the loss of water through steam, the glasses were covered with watch glasses with a diameter corresponding to the diameter of the glass. Each of the samples was boiled for 20–120 min. After being removed from the induction cooker, the resulting liquid was cooled, and the amber was taken out. After drying, the amber was weighed again, after which the difference in mass before and after evaporation was recorded. An Acculab ALC-210d4 laboratory analytical scale with a measurement error of 0.1 mg was used as an electronic scale. Some of the obtained values of the mass difference turned out to be less than the error of the instrument, so it was not possible to calculate the approximate concentration of the amber extract. However, in the study, we decided to test all the obtained samples in order to determine the success of the extraction of Baltic amber depending on the boiling time. The resulting samples are presented in Table 1.

Amber particles were investigated using the following parameters: panoramic acquisition was performed in the range of λ = 539–641 nm, averaging over measurements; the number of measurements—3; ND filter—0; and the accumulation time varied—10 s, 20 s, and 50 s. The amber extract was taken under the following conditions: panoramic acquisition at 539–641 nm, averaging over 3 measurements; ND filter—0; and accumulation time—30 s. In both cases of the study, a grating of 300 gr/mm was used. The obtained spectra were saved in the txt format, which was further processed in the Origin program. After processing, a spectral comparison of the experimental graphs with the KnowItAll (Willey, UK) database was carried out.

### 2.3. SERS Experiment

To implement the SERS technique, silver NPs were created in accordance with the method of chemical reduction from AgNO_3_ salt. When obtaining silver NPs by this method, the method of chemical reduction according to Turkevich [52], used for the chemical synthesis of silver and gold, was used. In 500 mL of distilled water, 50 mg of AgNO_3_ silver nitrate salt was dissolved. The solution was brought to a boil while being intensively stirred, after which 9 mL of a solution of aqueous sodium citrate, Na_3_C_6_H_5_O_7_, with a concentration of 1% was added to it. After thorough mixing, the solution changed color from transparent to yellow–green. Thus, silver NPs were reduced from the silver nitrate salt. The process of chemical reduction of silver corresponds to the following equation:

6AgNO_3_ + 3Na_3_C_6_H_5_O_7_ → 6Ag + 3Na_2_C_5_H_4_O_5_ + 3CO_2_ + 3NaNO_3_ + 3HNO_3_ (1)

The concentration of the resulting solution was calculated in accordance with the formula

(2) $$N=\frac{3m}{4\pi r^{3}\rho }$$

where *r* denotes the radius of silver particles, *m* = 50.1 mg denotes the mass of silver in solution, and ρ = 10.5 g/cm^3^ denotes the density of silver. The molar concentration *C_Ag_* of silver was determined according to the following expression:

(3) $$C_{Ag}=\frac{N}{N_{a}V}\;$$

The molar concentration of the colloidal silver solution used was *C* = 4.5 × 10^−^^10^ M.

**Figure 2:**
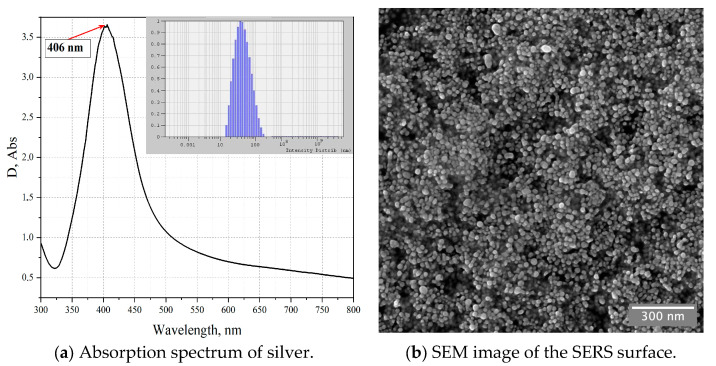
Absorption spectrum of silver NPs absorption at λ = 406 nm. Inset on the right: size distribution of silver NPs with a maximum at d = 44 nm.

The plasmonic absorption spectra and the size of silver nanoparticles were determined using a Shimadzu-2600 spectrophotometer and a Photocor Complex (LTD “Photocor”, Moscow, Russia) dynamic light scattering setup, respectively. The particle size was determined as d = 44 nm (inset in Figure 2). Based on the chosen data processing model, the integral relative measurement error was 0.0035%. The maximum plasmonic absorption was recorded at a wavelength of λ = 406 nm (Figure 2a). The high-resolution Scanning electron microscopy (SEM) has been used to produce secondary-electron images of synthetic samples of silver nanoparticles. All SEM images ware obtained on Zeiss Crossbeam 540 FIB-SEM system (Oberkochen, Germany), which is part of a unique scientific facility “SynchrotronLike” (Kaliningrad, Russia). The SEM image of silver nanoparticles (Figure 2b) was collected at 10 kV beam energy and 200 pA beam current (FOV ~ 1.6 x 1.6 um) with InLens (SE) electron detector. According to SEM, the average particle size was 17 ± 1 nm (the analysis was performed in ImageJ software using built-in “analyze particles” plugin). The difference between the Photocor and SEM data values is caused by the citrate shell of the nanoparticle stabilizer. In this case, according to the SEM data, we can discuss about the core (17 nm) of the particle and the shells around it (44 nm).

Since silver hydrosols have stable plasmon absorption in the visible region and their spectrum overlaps with the absorption and fluorescence spectra of amber, it was therefore possible to implement the processes of plasmon energy transfer in the amber/NP complex.

**Figure 3:**
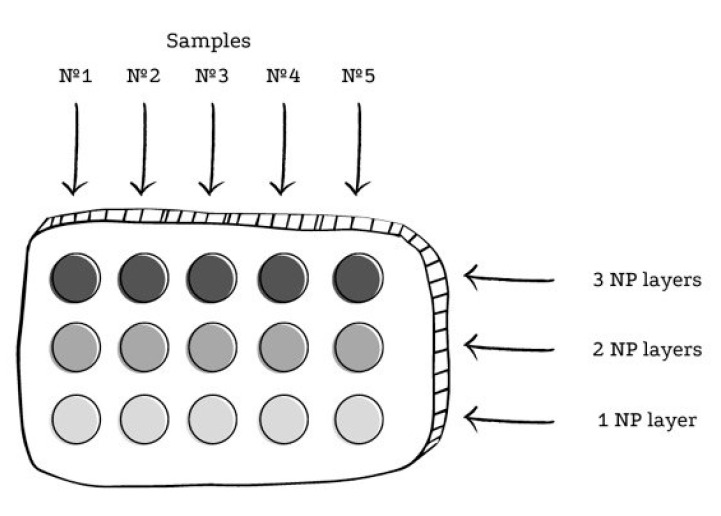
Scheme of the arrangement of samples on quartz glass during the experiment with an amber extract and silver NPs. A DPSS laser with a wavelength of λ = 532 nm and a power of 50 mW was used as a source of monochromatic radiation.

Registration of the SERS spectra of amber was carried out using the Centaur U HR research complex (LTD “NanoScanTechnology”, Moscow, Russia). Extracted amber was investigated with silver NPs via the following technique: Optically clear quartz glass was covered with 5 drops of 2 µL of colloidal silver in 3 rows. The quartz substrate was placed in a UT-4610 (LTD “UralSnab”, Izhevsk, Russia) oven for 5 min, where the citrate sol was dried at 38 °C. After surface drying, another 2 µL layer of silver citrate sol was applied on top of the first layer in drops. The quartz plate was dried again under the same conditions. After drying, another layer of sol was applied to the uppermost row, in drops of 2 μL. The quartz cuvette was dried again under the same conditions. After drying, a sample of 2 µL was applied to each of the island silver films using an Eppendorf Research automatic pipette with a tip. The quartz cuvette was again dried under the same conditions. After that, it was placed on the scientific unit holder for further investigation. The layout of samples on quartz glass during the experiment with an amber extract and silver NPs is shown in Figure 3.

Using a digital video camera IDus 401-DV CCD camera (Andor, UK) with a 1024 × 256 pixels sensor, an image was obtained from the sample. The image from the camera was displayed on a computer screen using the NSpec (LTD “NanoScanTechnology”) software (version 16.0), where the device parameters were adjusted, the opto-mechanical module, monochromator, and laser radiation source were controlled, data were controlled and received from detectors, data were processed, and samples were taken. Amber extract on silver island films was investigated under the following conditions: panoramic acquisition at λ = 539–641 nm, averaging over 3 measurements, ND filter—0, accumulation time—30 s, and a grating with 300 gr/mm. The obtained spectra were saved in the txt format and were further processed in the Origin program.

### 2.4. Simulation of Raman Spectra

**Figure 4:**
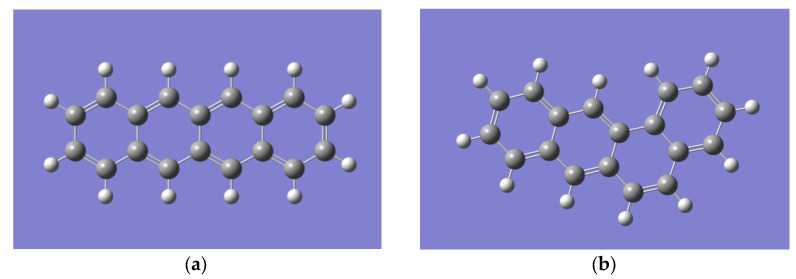
Molecules built in GaussView 6: (**a**) tetracene (naphthacene), (**b**) benzo [a]anthracene.

The Gaussian 16 software package (license number: G64284555249899W-6922N) was used to calculate the theoretical Raman spectra. In the GaussView 6 molecular visualization program, the structure of the substances analyzed in the Baltic amber was built: tetracene (naphthacene) (Figure 4a) and benzo [a]anthracene (Figure 4b).

The Raman spectra were obtained via the DFT method using the hybrid three-parameter Becke–Lee–Yang–Parr (B3LYP) exchange-correlation functional based on optimized molecular structures. As a basis set, a bivalent basis set with a split valence of 6–31G (d) was chosen, which includes 6 primitive Gaussians that make up the basis function of each base atomic orbital. The first valence orbital is made up of a combination of 3 primitive Gaussian functions, and the other is made up of a linear combination of 1 primitive Gaussian function. Based on the selected vibrational modes, an envelope line was constructed, the graph of which was used later to analyze the obtained experimental data.

The MP2 approximation was used to correct the obtained values for frequencies. As a criterion for assessing the quality of the calculation of vibrational frequencies, we used the value of the sum of squared deviations from a linear dependence.

### 2.5. FT-IR Experiment

To obtain FT-IR spectra, an IR-Prestige-21 (Shimadzu, Kyoto, Japan) spectrophotometer was used. The samples were prepared in KBr pellets. For this, the required amount of amber was weighed using electronic scales (AcuLab, Norwood, MA, USA). Amber pieces were placed in an agate mortar and crushed into crumbs as a next step. An amount of KBr powder, pre-weighed on an electronic balance, was added to the resulting crumb. Crushed amber was mixed with potassium bromide. After that, the agate mortar with the sample was placed in the UT-4610 oven for 10 min at 36 °C to dry the moisture that the potassium bromide powder could absorb to avoid the appearance of water absorption bands in the spectrum. After drying, the sample was rubbed again.

Samples with silver NPs were prepared as a second step. With the help of electronic scales, the required amount of amber was weighed. This amount was placed in an agate mortar and crushed into crumbs as the next step. A certain amount of KBr powder pre-weighed on an electronic balance was added to the resulting crumbs. Crushed amber was mixed with potassium bromide. After that, the agate mortar with the sample was placed in the UT-4610 oven for 10 min at 36 °C to dry the moisture. After drying, some colloidal silver was added to the sample using an Eppendorf Research automatic pipette with a tip and triturated again. After that, the agate mortar with the mixture was again placed in the UT-4610 oven for 10 min at a temperature of 36 °C to dry out the moisture. After that, the mixture was ground again and again placed in a UT-4610 oven under the same conditions. The resulting samples are presented in Table 2.

## 3. Results and Discussion

### 3.1. Spectrophotometry of Amber

**Figure 5:**
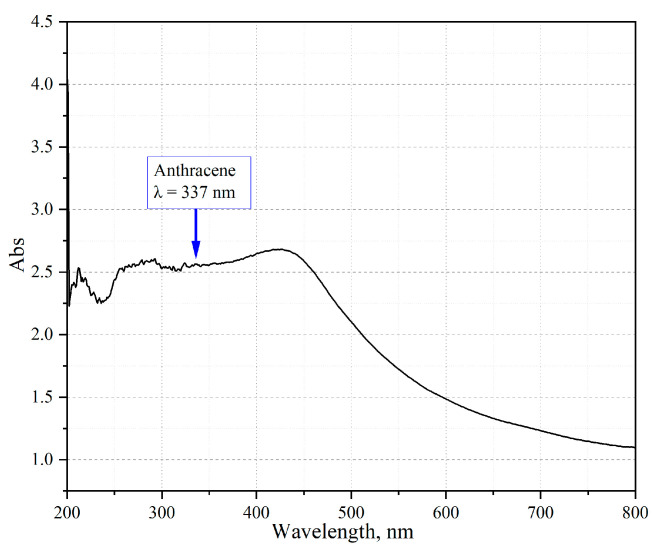
Absorption spectrum of Baltic amber in the wavelength range 200–800 nm.

Since amber has a rather complex structure, the first stage of the study was the interpretation of the data on its absorption components (Figure 5).

As can be seen from the resulting spectrum, Baltic amber absorbs in a wide range of wavelengths, from the UV region to the near IR region, which is ensured by the complexity of the structure and the presence of several absorbing components. Several maxima are distinguished in the spectrum of amber. It is difficult to determine which aromatic compounds they belong to due to the complex structure of amber. The maximum absorption value of Baltic amber can be observed when amber is irradiated with a wavelength of λ = 423 nm. As the wavelength increases further, the absorption spectrum decreases monotonically. According to the literature data [53], a fairly wide range of compounds in amber has been identified (Acenaphthene, 1-Methylnaphthalene, Phenanthrene, Anthracene, 2,6-Dimethyl-naphthalene, 1,6-Dimethyl-naphthalene, Reten (1-methyl-7-isopropylphen- anthrene), 2-Methyl-anthracene, 2,3-Dimethyl-naphthalene, 9-Methylnaphthalene, Phenanthrene) with the most intense absorption maximum of close derivatives of anthracene (Anthracene, Methyl-anthracene, Tetracene), with which further work was carried out.

### 3.2. Amber Particles

The research was carried out using Raman and SERS spectroscopies of amber particles and extracts. The most intense vibrations for particles were detected at 1141 cm**^−^**^1^, 1647 cm^−^^1^, 2876 cm^−^^1^, and 2939 cm^−^^1^.

**Figure 6:**
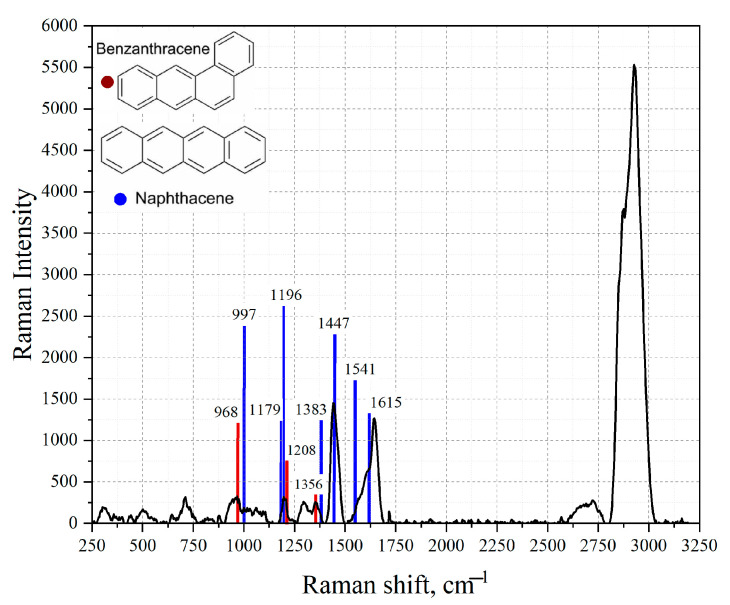
Raman scattering spectra of amber particles. The blue lines show the position of the peaks characteristic of the tetracene molecule. The red lines show the position of the peaks characteristic of the benzoanthracene molecule.

In the analyzed spectra, vibrations related to the structure of tetracene and benzo [a]anthracene are observable. Based on the studies of the Raman spectra of the studied substances, the following vibrational modes for tetracene can be distinguished [54]: C-H bending vibrations at 1196 cm^−^^1^, C-C stretch oscillation at 1447 cm^−^^1^, and C-C stretching at 1615 cm^−^^1^. For benzanthracene on the obtained spectrum of Baltic amber crumbs, characteristic fluctuations at 968 cm^−^^1^, 1208 cm^−^^1^, and 1356 cm^−^^1^ can be distinguished [53]. To analyze the amber structure in greater depth, we used the FRET theory based on the spectral overlap of tetracene and benzanthracene fluorescence with a silver absorption band. This made it possible to effectively transport plasmon energy in the complex and study tetracene components in detail. Figure 6 shows the fingerprint region of the Raman spectrum of amber particles with the selected vibration modes characteristic of aromatic compounds.

### 3.3. Amber Extracts

One of the solutions for isolating fluorescent groups in the structure of amber is their extraction. When amber is heated, a coniferous smell can be detected, which indicates the release of some aromatic groups. But it is difficult to collect vapors emanating from a solid sample. One way to study the fumes that come out of amber when it is heated is to boil the amber. Thus, all chemical compounds will remain in the liquid, which can be studied using Raman spectroscopy and SERS.

**Figure 7:**
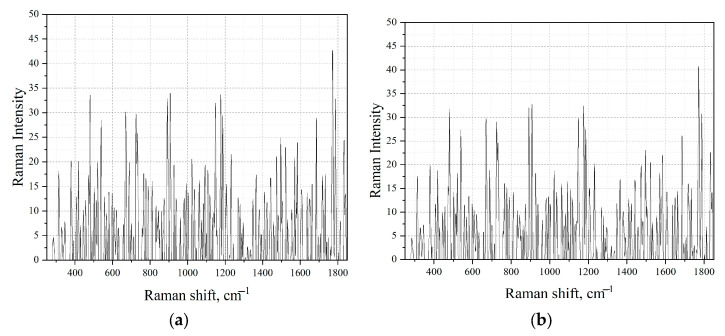
Raman spectra of amber extracts with (**a**) the longest boiling time t = 120 min, and (**b**) the shortest boiling time t = 20 min.

The next stage of the study was to obtain the Raman spectra of the obtained amber extracts with different boiling times. However, in the course of the study, it was found that the obtained extracts of amber, regardless of the boiling time, cannot be detected using Raman spectroscopy (Figure 7).

**Figure 8:**
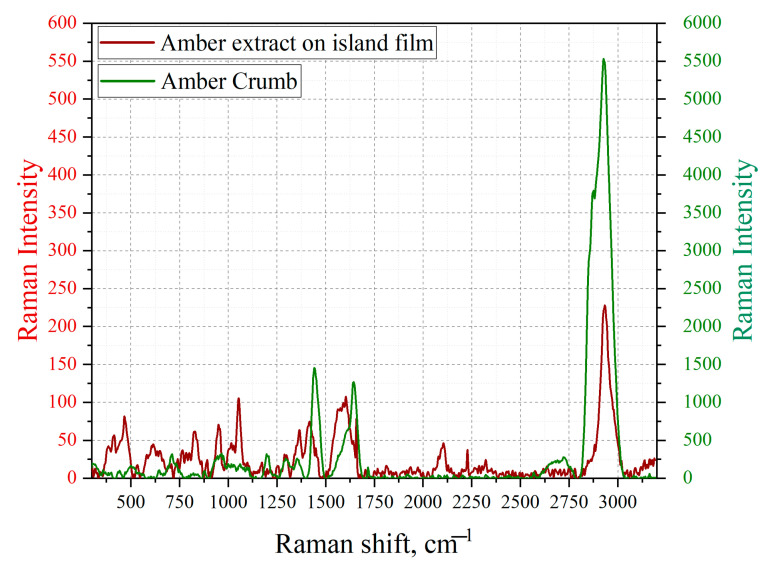
Comparison of the Raman spectrum of amber particles (green graph, *y*-axis on the right) and SERS of amber extract (red graph, *x*-axis on the left).

Since the obtained spectra were low intensity and highly noisy, it was not possible to spectrally identify the extracted aromatic compounds. To confirm the hypothesis that extraction makes it possible to isolate aromatic compounds from the structure of Baltic amber, the spectral identification of this compound was carried out using the SERS method (Figure 8).

**Figure 9:**
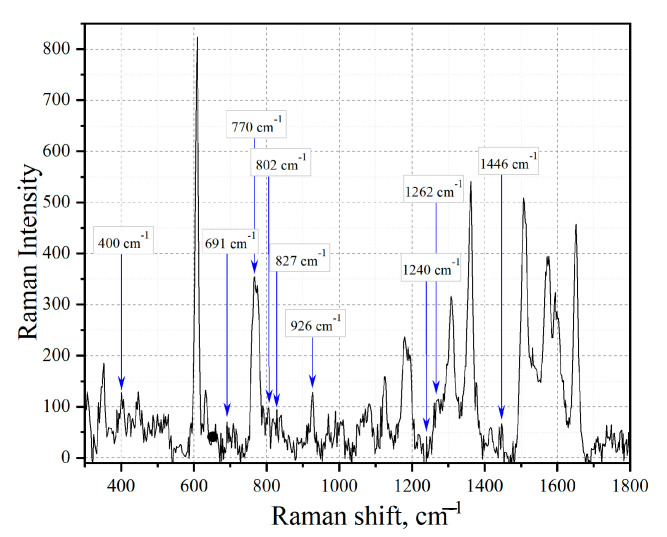
SERS spectrum of amber extract with the shortest boiling time t = 20 min on one layer of a silver island film.

**Figure 10:**
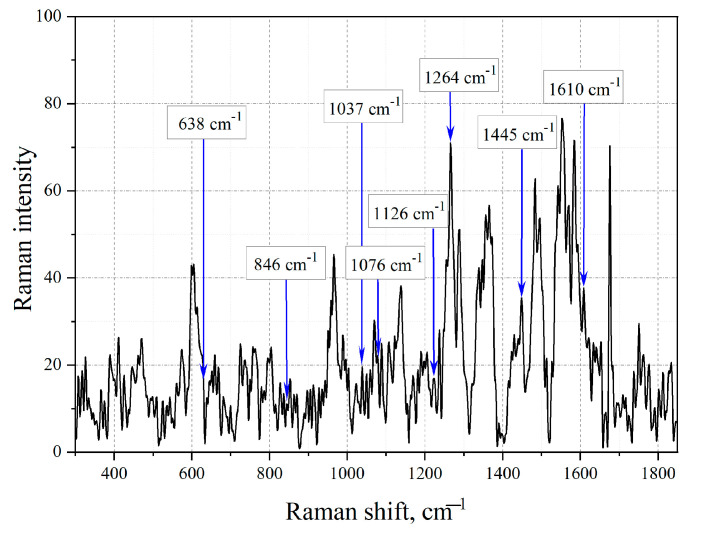
Extract obtained at boiling time t = 80 min on a 3-layer island film.

Figure 8 demonstrates that the extraction of amber by evaporation of aromatic compounds from the natural resin was successful, as evidenced by the maximum at 2939 cm^−^^1^, which characterizes the deformation of the aromatic ring. Therefore, in the future, we will consider the fingerprint zone in the SERS spectra of extracted amber (Figure 9 and Figure 10).

Using the KnowItAll program, the vibration modes of the analyzed sample were determined. Table 3 shows the maximums shown by the samples.

Using the KnowItAll program, the vibration modes of the analyzed sample were determined. Table 4 shows the maximums shown by the samples.

Examining the extracted amber, it is observable that the vibrations identified earlier in the structure of natural resin for tetracene (1196 cm^−^^1^, 1447 cm^−^^1^, 1615 cm^−^^1^) [53] and for benzanthracene (968 cm^−^^1^, 1208 cm^−^^1^, 1356 cm^−^^1^) [55] have been preserved, which allowed us to conclude that these chemical compounds were successfully extracted.

The Raman spectrum of an amber extract without silver NPs could not be analyzed due to strong noise due to the weak response of the sample to radiation. However, when investigating an amber extract, a resolved spectrum appears on silver island films, which makes it possible to analyze the compound and, in particular, to detect tetracene and benzo [a]anthracene, despite the fact that the observed signal enhancement is small.

The EF SERS coefficient can be calculated using the formula:

(4) $$K_{SERS}=\frac{I_{SERS}\cdot \;C_{Raman}}{I_{Raman}\cdot \;C_{SERS}}$$

where *I_SERS_* and *I_Raman_* denote the spectral intensities in the presence and absence of NPs, respectively, and ***C****_Raman_* and ***C****_SERS_* are the concentration of the test sample without NPs and with NPs, respectively. If the equal concentration for SERS and RS was used, Formula (4) can be simplified:

(5) $$K_{SERS}=\frac{I_{SERS}}{I_{Raman}}$$

The EF SERS coefficient was calculated for the concentration of amber extract at t = 120 min and *K_SERS_* = 30.

### 3.4. Simulation of Extract Spectra

**Figure 11:**
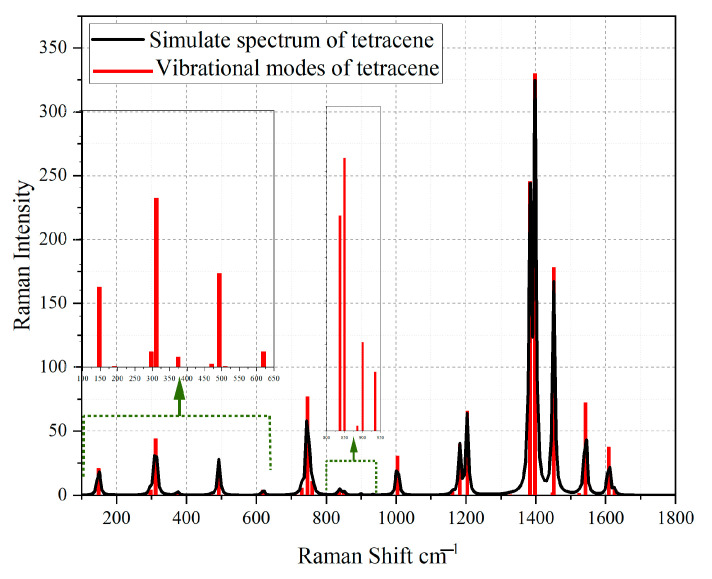
Simulated spectrum of the tetracene molecule. The red bands represent the simulated vibrational modes of tetracene. The black graph is the envelope of vibrational modes. The graph shows fluctuations of low intensity in the indicated interval.

**Figure 12:**
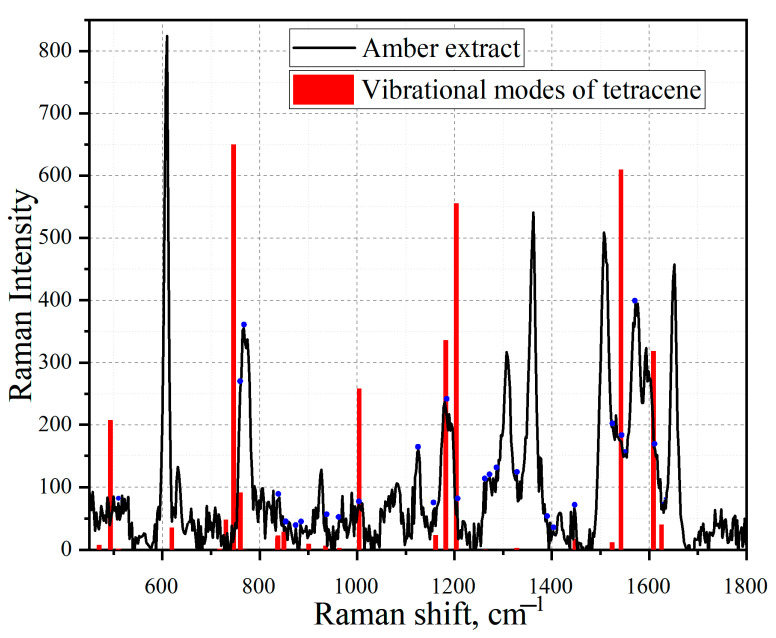
Comparison of the SERS spectrum of the extract of amber with boiling time t = 80 min on one layer of silver island film (black graph) and the simulated vibrational modes of tetracene (red line). The blue dots mark vibrations corresponding to the molded structure of tetracene. In order to consider low-intensity oscillations, high-intensity oscillations were removed from the comparison graph but taken into account when vibrational modes were required.

**Figure 13:**
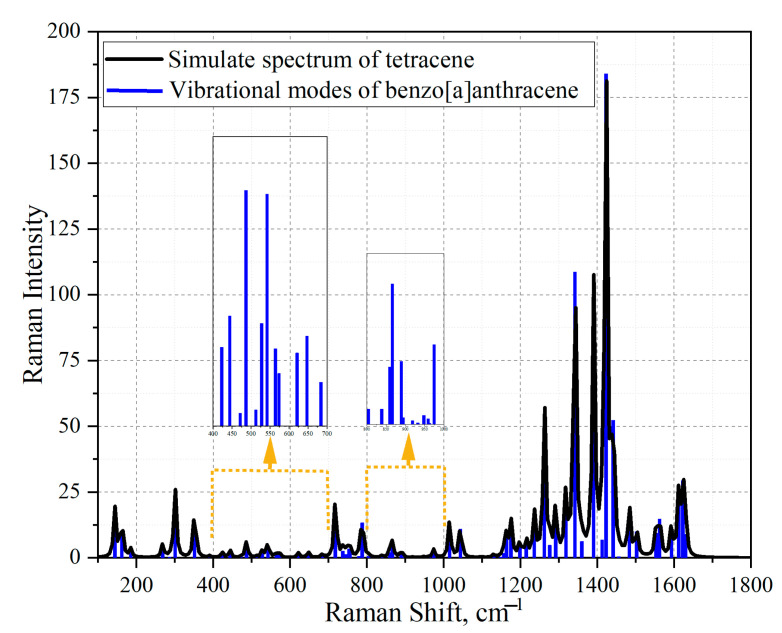
Modeled spectrum of the benzanthracene molecule. The blue bands represent the simulated vibrational modes of benzanthracene. The black graph is the envelope of vibrational modes. The graph shows fluctuations of low intensity in the indicated interval.

**Figure 14:**
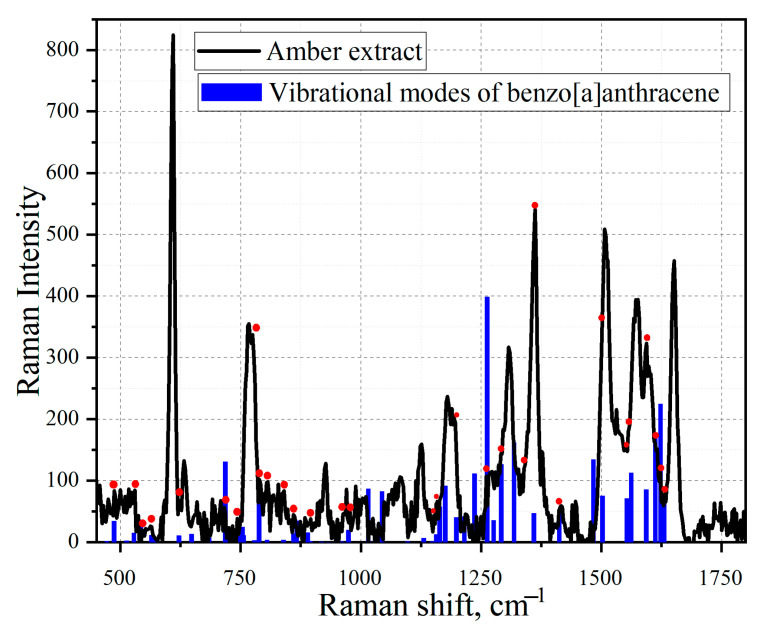
Comparison of the SERS spectrum of extract of amber with boiling time t = 20 min on one layer of silver island film (black graph) and the simulated vibration modes of benzanthracene (blue line). The red dots mark vibrations corresponding to the molded structure of benzanthracene.

Using the Gaussian software, the mathematical modeling of the substances tetracene and benzo [a]anthracene was carried out and confirmed. Next, the simulated spectra of tetracene (Figure 11) and the comparison of the vibration modes of tetracene on the spectrum of an amber extract (Figure 12) were presented. The spectrum of benzanthracene was also modeled (Figure 13). The vibration modes of this compound were also compared with the SERS spectrum of the amber extract (Figure 14).

When comparing the practical graph of the amber extract with the theoretical graph of tetracene, several corresponding fluctuations can be distinguished, which are presented in Table 5.

When comparing the practical graph of the amber extract and the theoretical graph of benzanthracene, several corresponding fluctuations can be identified, which are presented in Table 6.

### 3.5. FT-IR Spectroscopy

**Figure 15:**
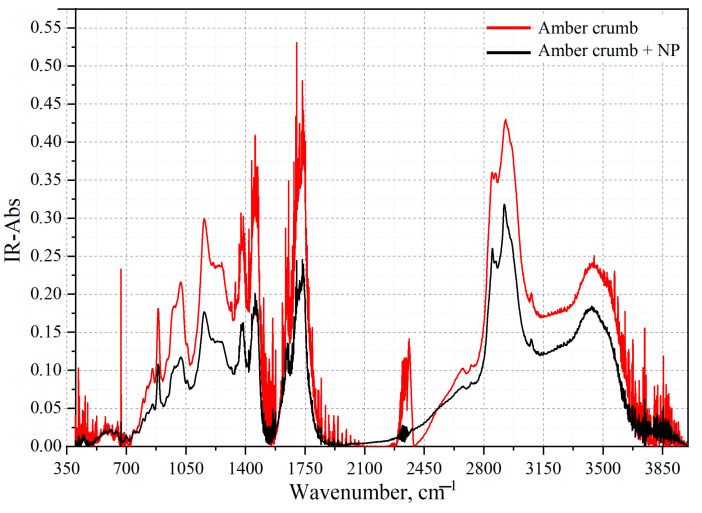
Comparison of the FT-IR spectrum of amber crumb without NP (red line) and FT-IR spectrum of amber crumb with 3 μL NP.

A comparison of the obtained FT-IR spectra of amber chips with and without NPs is shown in Figure 15.

Analyzing the graphs, it can be seen that without NPs, a large amount of noise appears in the sample, which complicates the process of determining the substance included in the sample. However, the addition of a small number of silver NPs (3 μL) reduces the amount of noise, which improves the possibility of detecting the sample and determining the vibrational structures contained in them. In this regard, we can conclude that there is a use for a plasmon-enhanced IR spectroscopy technique. For amber chips, several IR absorption maxima are observed, corresponding to OH stretching vibrations: a maximum at 3424 cm^−1^ and a maximum at 1381 cm^−1^. The maximum at 2920 cm^−1^ corresponds to asymmetric C-H stretching vibrations. The maximum at 1120 cm^−1^ corresponds to asymmetric and symmetric C-O-C stretching vibrations. The maximum at 890 cm^−1^ corresponds to fan-shaped C-H stretching vibrations [56,57].

It should be noted that amber extracts with different boiling times were studied via FT-IR spectroscopy. However, regardless of the number of added NPs, the FT-IR spectrum failed to detect the amber extract. Therefore, metal-enhanced IR spectroscopy is not suitable for this study.

## 4. Conclusions

As a result of the paper, it was possible to isolate amber’s fluorescent components using the extraction method. Using SERS, it was possible to confirm anthracene derivatives in amber, namely tetracene and benz[a] nthracene. The extraction of amber can help distinguish the chemical structure of different kinds of amber and, consequently, the deposit and its value. With the usage of silver nanoparticles, SERS methods for the study of amber have been successfully implemented. It has been shown that SERS methods are effective for aromatic compound detection, increasing the registered signal by at least 30 times. Using the method of mathematical modeling, anthracene derivatives were modeled, and their presence in the structure of Baltic amber was shown. The purposes for differentiation for ambers were shown.

## Figures and Tables

**Table 1:** Parameters of the study of the amber extract using Raman spectroscopy.

Mass of Amber before Evaporation, g	Volume of Water, mL	Boiling Time, min	Mass of Amber after Evaporation, g	Mass Difference of Amber before and after Evaporation, g
0.5505	40	20	0.5503	0.0002
0.5694	40	40	0.5689	0.0005
0.5334	40	60	0.5318	0.0016
0.5450	40	80	0.5441	0.0009
0.5486	40	120	0.5467	0.0019

**Table 2:** Parameters of the study of the amber crumb using FT-IR spectroscopy.

Amber Mass, g	KBr Powder Mass KBr, g	Volume of Silver NPs, mkl	The Mass of the Mixture Taken for Pressing, g
0.0375	0.4353	-	0.1576
0.0336	0.4200	3	0.1512
0.0343	0.4206	5	0.1527
0.0347	0.4232	10	0.1536

**Table 3:** Raman spectra characteristics of Amber extract with the shortest boiling time t = 20 min on one layer of a silver island film.

Group (Aromatics)	Bond	Spectral Shift Position, cm^−1^	Intensity	Mode
o-disubstituted ring	CH	1262	weak	bending in-plane H bend
	CH	1126	weak	bending in-plane H bend
	CH	926	weak	four adjacent H out-of-plane deformations
	CH	827	weak	five adjacent H out-of-plane deformations
p-disubstituted ring	Ring	1446	medium	stretching
	CH	1117	weak	bending in-plane H bend
1,3,5-trisubstituted	Ring	1446	medium	stretching
	CH	1262	weak	bending in-plane H bend
	Ring	705	strong	out-of-plane ring bending
1,2,3,4,5,6-substituted	Ring	1446	medium	stretching
	Ring	400	medium–strong	deformation
m-disubstituted ring	Ring	1446	medium	stretching
	Ring	691	strong	out-of-plane ring bending
1,2,4,5-substituted	Ring	1446	medium	stretching
	CH	802	weak	one adjacent H out-of-plane deformation
1,2,4-trisubstituted	CH	770	strong	two adjacent H out-of-plane deformations

**Table 4:** Raman spectra characteristics of amber extract with boiling time t = 80 min on a 3-layer island film.

Group (Aromatics)	Bond	Spectral Shift Position, cm^−1^	Intensity	Mode
o-disubstituted ring	Ring	1602	Medium	bending–stretching
	Ring	1577	Medium	bending–stretching
	Ring	1445	Medium	stretching
	CH	1264	Medium	bending in-plane H bend
	CH	1126	Weak	bending in-plane H bend
	CH	1076	Weak	bending in-plane H bend
	CH	1037	Weak	bending in-plane H bend
p-disubstituted ring	Ring	1610	Medium	bending–stretching
	Ring	1503	Weak	bending–stretching
	CH	981	Weak	bending in-plane H bend
	CH	638	Weak	bending in-plane H bend
1,3,5-trisubstituted	Ring	1610	Medium	stretching
	Ring	1498	Medium	stretching
	Ring	1265	Weak	bending in-plane H bend
	Ring	1015	Strong	bending in-plane H bend
1,2,3,4,5,6-substituted	Ring	1610	medium–strong	stretching
1,2,3,4,5-substituted	CH	875	Weak	one adjacent H out-of-plane deformation
1,2,3,4-substituted	Ring	1445	Medium	stretching
	CH	846	Weak	two adjacent H out-of-plane deformations
1,2,3,5-substituted	Ring	1610	Medium	stretching
	Ring	1498	Weak	stretching
	Ring	1445	Medium	stretching
	CH	846	Weak	one adjacent H out-of-plane deformation
1,2,3-trisubstituted	Ring	1610	Medium	stretching
	Ring	1498	Weak	stretching
	Ring	1445	Weak	stretching
	CH	1155	Weak	weak
	CH	1076	Weak	bending in-plane H bend
	CH	1019	medium–strong	bending in-plane H bend
m-disubstituted ring	Ring	1610	medium–weak	bending–stretching
	Ring	1233	medium–strong	bending in-plane H bend
	CH	1159	Weak	bending in-plane H bend
1,2,4,5-substituted	Ring	1610	Medium	stretching
	Ring	1498	Weak	stretching
	CH	1193	Weak	bending
	CH	886	Weak	one adjacent H out-of-plane deformation
1,2,4-trisubstituted	Ring	1610	Medium	stretching
	Ring	1583	Medium	stretching
	Ring	1498	Weak	stretching
	Ring	1445	Weak	stretching
	CH	1209	Weak	bending
	CH	1167	Weak	bending
	CH	1027	Weak	bending

**Table 5:** Modes isolated in the structure of tetracene, correlated with the structure of the amber extract with boiling time t = 80 min.

Group (Aromatics)	Bond	Spectral Shift Position, cm^−1^	Intensity	Mode
1,2,3,4-substituted	CH	846	Weak	two adjacent H out-of-plane deformations
1,2,3,5-substituted	CH	846	Weak	one adjacent H out-of-plane deformation
1,2,3,4,5-substituted	CH	875	Weak	one adjacent H out-of-plane deformation
1,2,4,5-substituted	CH	886	Weak	one adjacent H out-of-plane deformation
o-disubstituted ring	CH	1125	Weak	bending in-plane H bend
1,2,4-trisubstituted	CH	1165	Weak	bending
1,2,4,5-substituted	CH	1183	Weak	bending
1,2,3,4-substituted	CH	1204	Weak	bending
1,2,4-trisubstituted	CH	1206	Weak	bending
o-disubstituted	CH	1260	Medium	bending in-plane H bend
1,3,5-trisubstituted	Ring	1265	Weak	bending in-plane H bend
1,2,3-trisubstituted	Ring	1445	Weak	stretching
m-disubstituted ring	Ring	1610	medium–weak	bending–stretching

**Table 6:** Modes isolated in the structure of benzanthracene, correlated with the structure of extract of amber with boiling time t = 20 min.

Group (Aromatics)	Bond	Spectral Shift Position, cm^−1^	Intensity	Mode
1,2,4-trisubstituted	CH	770	Strong	two adjacent H out-of-plane deformations
1,2,4,5-substituted	CH	805	Weak	one adjacent H out-of-plane deformation
o-disubstituted ring	CH	1262	Weak	bending in-plane H bend

## Data Availability

Data is contained within the article.
